# The Application of Eight-Segment Pulmonary Rehabilitation Exercise in People With Coronavirus Disease 2019

**DOI:** 10.3389/fphys.2020.00646

**Published:** 2020-05-29

**Authors:** Jian-Min Chen, Zhi-Yong Wang, Yang-Jia Chen, Jun Ni

**Affiliations:** Department of Rehabilitation Medicine, The First Affiliated Hospital of Fujian Medical University, Fujian, China

**Keywords:** COVID-19, pneumonia, pulmonary rehabilitation, Health Qigong, proprioceptive neuromuscular facilitation

## Introduction

A novel coronavirus, now named COVID-19 by the World Health Organization (WHO), broke out in Wuhan in December 2019 and spread rapidly to other provinces in China, as well as to many countries (Zhu et al., 2020). More than 80,000 patients were diagnosed with COVID-19 in China. COVID-19 is an infectious disease caused by a novel coronavirus, and according to traditional Chinese medicine (TCM), the pathology of COVID-19 is considered to be within the scope of “plague” (Yang et al., 2020), which leads to varying degrees of respiratory dysfunction in patients based on their age, physical state, and underlying diseases before being infected by the virus. Among patients with COVID-19, some may develop acute respiratory distress syndrome (Huang et al., 2020). Fortunately, most patients only suffer mild symptoms, including fever, cough, and myalgia or fatigue (Fu et al., 2020). The first pathology report on COVID-19 demonstrated that the disease has severe impacts on the patient's lungs, hearts, and other tissue, as illustrated by autopsy reports (Liu et al., 2020). So far, there are no effective antiviral drugs against the coronavirus, and determining how to relieve the clinical symptoms of these patients, promote lung function recovery, and reduce the fatality rate is still an urgent unsolved problem (Sun et al., 2020). Most patients with COVID-19 have been subjected to isolation as a management strategy; however, during “isolation,” limited activity space and unintentional lengthening of inactive time will lead to a significant reduction in level of physical activity (Brugliera et al., 2020). Recent reports have suggested the safety and effectiveness of rehabilitation intervention for patients with COVID-19 (Brugliera et al., 2020; Simpson and Robinson, 2020). Early adequately moderated rehabilitation strategies are needed to promote recovery from pneumonia and interrupt this unfavorable cycle of reduced physical activity, functional capacity decline, worsening symptoms, and increased risk of secondary complications (Blondeel et al., 2018). Therefore, the First Affiliated Hospital of Fujian Medical University designed an exercise program, the “Eight-segment pulmonary rehabilitation exercise” to improve the condition of patients with COVID-19 with the aim of promoting recovery of their pulmonary and physical function.

## The Exercise As A Viable Rehabilitation Option

The Eight-Segment Pulmonary Rehabilitation Exercise is based on a combination of selected “routines” from Health Qigong (HQG), part of traditional Chinese medicine, together with exercise training of pulmonary rehabilitation and proprioceptive neuromuscular facilitation (PNF), techniques belonging to Western medicine.

Pulmonary rehabilitation is an effective non-drug intervention that includes physical exercise training, encouraging behavioral change, and stimulus to physical activity (Dowman et al., 2014; Mendes et al., 2019); it has been proven to be beneficial for people with most lung conditions (Cancelliero-Gaiad et al., 2014; Stephens et al., 2017). The pathophysiology of COVID-19 may be limitation of ventilation and diffusion dysfunction (Xu et al., 2020), making it similar to that of severe acute respiratory syndrome CoV (SARS-CoV) (Fu et al., 2020). Patients may achieve the goals of increasing peripheral muscle performance and strengthening respiratory muscles, expectoration, lung compliance, and vital capacity through pulmonary rehabilitation exercise training (Dowman et al., 2014).

Proprioceptive neural facilitation (PNF) is an elemental physiotherapeutic strategy (Gong, 2020) that is associated with specific movement patterns in diagonal and spiral directions (Areas et al., 2013; Slupska et al., 2019). Physiotherapists frequently use this technique to increase muscle intensity, proprioception, active range of motion, and motor control (Hindle et al., 2012; Sayaca et al., 2020). Due to the disease and reduced activity, the respiratory muscles of patients with COVID-19 are weakened. PNF could help to improve respiratory muscular performance in order to achieve the goals of normalizing breathing pattern and improving lung ventilation and thus tissue oxygenation (Areas et al., 2013; de Souza et al., 2020).

In addition, Chinese people, especially the elderly, have a strong preference and high acceptance of HQG due to their cultural background. HQG is associated with various forms of cultural sporting activities that use the principles of TCM and is reported to be beneficial for patients with lung diseases (Ng et al., 2014). Through the movement of the extremities, meditation, breathing control, and other forms in Qigong, it has been shown to promote the exercise capacity and respiratory function of patients with Novel Coronavirus Pneumonia (NCP) to a certain extent (Ding et al., 2014). In addition, Qigong could confer an anti-inflammatory effect in patients with obstructive ventilation dysfunction diseases, such as chronic obstructive pulmonary disease (COPD). However, whether HQG has any effect on people with COVID-19 needs further research (Ng et al., 2014).

Subjects fulfilling the following criteria were recruited to the exercise group: (1) age range from 18 to 75 years; (2) a COVID-19 severity level belonging to the mild to moderate stages according to the COVID-19 Treatment Guidelines from China; (3) the muscle strength of all limbs being greater than Grade 4 according to manual muscle testing; (4) a body mass index between 18.5 and 29.99 kg/m. Exclusion criteria included: (1) other severe respiratory or systemic diseases critical conditions; (2) a Montreal Cognitive Scale score of <26; (3) other safety contraindications to exercise.

Two investigators (LL and YZZ) were previously trained to carry out data collection, and they provided the instructions to the participants. This study was approved by the ethics committee of the institution (MRCTA, ECFAH of FMU[2020]038), and all the participants signed a written consent form.

As well as standard care (nutrient Supplementary Material, drug therapy, oxygen, Chinese Traditional Medicine, etc.), each subject received an extra 30-min exercise session led by a trained rehabilitation therapist every day during hospitalization for 2 weeks, with a learning package in the form of audio and visual materials. Until 4 weeks of follow-up after discharge, they were required to continue the program by using the materials, which can be viewed on a tablet, mobile phone, or networked TV. The exercise protocol consisted of eight full-body low-intensity distinct sections (Supplementary Materials 1, 2), with each section being repeated six times. The entire protocol usually took 10–12 minutes to complete at the usual pace, and the training was repeated 2 times, separated by a few minutes break (Ng et al., 2011; Liu et al., 2012). To address the special needs of patients with COVID-19, an expert panel review was conducted to assess the potential clinical risk of this rehabilitation exercise. Tests were also conducted in isolation wards and home environments to study the safety in its application. To ensure safety, patients could maintain nasal cannula oxygen during training and immediately stop training if symptoms such as fatigue and asthma occurred. To keep a record of their own practice, each patient was issued a daily log (Ng et al., 2011).

The integration of video and direct supervision was enjoyable for the subjects. The first pilot study consisted of 10 subjects who took the exercise and used it for 14 days under the guidance of therapists. The purpose was to test the feasibility and usability of the program. The demographic and clinical characteristics of these patients are shown in Table 1. The subjects were 100% adherent, reported that the program was easy to master, and, ultimately, adhered to it. In addition, post-program evaluation showed initial signs of improvement in physical activity, perceptions of dyspnea, and quality of life in these subjects when compared to a standard-care control group. However, this research is mainly an explanation of this exercise method, and standardized clinical research will be carried out immediately. As this exercise required very limited supplies and can be done almost everywhere, we believe that it could benefit a large portion of patients with COVID-19; therefore, we conclude that this exercise is to be recommended for COVID-19.

**Table 1:** Demographic and clinical characteristics of the 10 patients.

**Characteristic**	**Subjects (*n* = 10)**
Age (year)	50.2 ± 12.04
Male/female	6/4
Body mass index before exercise (kg/m 2)	23.45 ± 2.65
<20	2
≥20–25	4
≥25–30	4
Length of hospital stay (day)	5.0 ± 2
Disease stage	
Mild	2
Middle	8

## Conclusion

Nonetheless, it will be interesting to see whether this rehabilitation program is able to successfully promote the recovery of patients COVID-19 and improve the physical activity levels of patients. The authors are seeking to make the program accessible to more patients; this study furthers that important work. Of course, the program is a representative slice of a broader research effort. Further borrowing of aspects from different study designs and experiences are needed to advance the exploration of how to conduct rehabilitation training safely and effectively during the epidemic situation.

## Author Contributions

J-MC wrote the article. Z-YW and JN conceived the idea. Y-JC participated in designing the exercise.

## Conflict of Interest

The authors declare that the research was conducted in the absence of any commercial or financial relationships that could be construed as a potential conflict of interest.

## Supplementary Material

Supplementary Material 1 Eight-segment pulmonary rehabilitation exercise.

Supplementary Material 2 Selected routines.
