# Supplementary material for: The Application of Eight-Segment Pulmonary Rehabilitation Exercise in People With Coronavirus Disease 2019
Source: Front Physiol. 2020 May 29;11:646. doi: 10.3389/fphys.2020.00646 (PMC7273974; doi:10.3389/fphys.2020.00646)
Supplement: Supplementary Material 1 — Eight-segment pulmonary rehabilitation exercise. [file Table_1.DOCX]

**Eight-segment pulmonary rehabilitation exercise**

**Preparation type：**

Deep breathe and hold it at the pubic region.

Sequence of operation: From a standing position with feet shoulder-width apart, keep your hands folded before the abdomen and eyes looking straight ahead. Keep your lower lumbar straight and levator the anus. The lower abdomen plumps up and sucks in when inhaling and exhaling. Repeat the breath thrice, and have a rest.

**Section 1(stand high and look far)**

From a standing position with feet shoulder-width apart with arms slightly abduction, stand on your toes slowly while keeping hands in clenched fist. Hold for two seconds and then lower your heels slowly, open your hands slowly. Breathing points: Inhaling when standing on toes and hands fisted, exhaling when lowering heels.

**Section 2 (reverberate like thunder)**

From a standing position with feet shoulder-width apart, cross your hands, clench your fists and then touch your ears with your palms facing inward. Open and turn your hands slowly, palms facing out and down. Breathing points: inhaling when touching ears, exhaling when putting hands down.

**Section 3 (Tiannv Sanhua)**

Feet open with shoulder width. Cross your hands and place your fists in front of your abdomen with your palms facing each other. Open slowly upwards with palms up. Breathing points: Exhale when hands are crossed, inhale when slowly raised.

**Section 4 (Yangtian Changxiao)**

Open your feet slightly wider than your shoulders, squat with your legs a quarter, sit back with your hips, bend over and arch your head down, place your hands in front of the knees with your hands crossed and your fists. Slowly stand upright with your hands open outwards, lean your torso back, palms up. Breathing points: Exhale when squatting and bending, pay attention to the rectum, and inhale when slowly lifting.

**Section 5 (Liangjian Chuqiao)**

Feet open with shoulder width. With one hand on hips and the other with fists, bend over, and touch the back of your hand against the opposite knee. Torso rotates slowly open outwards with palms up. Breathing points: Exhale when squatting and bending, pay attention to the rectum, and inhale when slowly lifting Repeat this action on the opposite side.

**Section 6 (Light as a Swallow)**

Feet open with shoulder width Hands on hips One side of the lower limb is lifted to perform a shuttlecock action, and the ankle is stretched and taut.  Slowly straighten your legs to the back and outside, and point your toes Breathing points: Exhale when raising leg, inhale when slowly falling Repeat this action on the opposite side Elderly people or hand supporters with poor balance function can complete the above actions.

**Section 7 (Back to basics)**

Feet open with shoulder width and hands on hips. Raise one thigh to the outside, while the calf and ankle are pulled back and taut. Slowly stretch your legs forward and inward, with your toes pointed (beyond the midline). Breathing points: Exhale when raising leg, inhale when slowly falling Repeat this action on the opposite side Elderly people or hand supporters with poor balance function can complete the above actions.

**Section 8 (Swagger)**

Open your arms and relax naturally. Keep breathing naturally. Alternate lower leg movements.The torso and head rotate to the opposite side, and the arms naturally swing.
